# How Useful Are Monogenic Rodent Models for the Study of Human Non-Alcoholic Fatty Liver Disease?

**DOI:** 10.3389/fendo.2016.00145

**Published:** 2016-11-16

**Authors:** Jake P. Mann, Robert K. Semple, Matthew J. Armstrong

**Affiliations:** ^1^Department of Paediatrics, University of Cambridge, Cambridge, UK; ^2^The University of Cambridge Metabolic Research Laboratories, Wellcome Trust-MRC Institute of Metabolic Science, Cambridge, UK; ^3^The National Institute for Health Research Cambridge Biomedical Research Centre, Cambridge, UK; ^4^Centre for Liver Research, National Institute for Health Research (NIHR) Birmingham Liver Biomedical Research Unit, University of Birmingham, Birmingham, UK; ^5^Liver Unit, Queen Elizabeth University Hospital Birmingham, Birmingham, UK

**Keywords:** steatosis, animal model, metabolic syndrome, steatohepatitis, genetic models

## Abstract

Improving understanding of the genetic basis of human non-alcoholic fatty liver disease (NAFLD) has the potential to facilitate risk stratification of affected patients, permit personalized treatment, and inform development of new therapeutic strategies. Animal models have been widely used to interrogate the pathophysiology of, and genetic predisposition to, NAFLD. Nevertheless, considerable interspecies differences in intermediary metabolism potentially limit the extent to which results can be extrapolated to humans. For example, human genome-wide association studies have identified polymorphisms in PNPLA3 and TM6SF2 as the two most prevalent determinants of susceptibility to NAFLD and its inflammatory component (NASH), but animal models of these mutations have had only variable success in recapitulating this link. In this review, we critically appraise selected murine monogenic models of NAFLD, NASH, and hepatocellular carcinoma (HCC) with a focus on how closely they mirror human disease.

## Introduction

Non-alcoholic fatty liver disease (NAFLD) is a pandemic disorder associated with premature morbidity and mortality. Its etiology is multifactorial, with both genetic predisposition and environmental factors playing important parts (1). Animal models have been central to recent major translational research efforts, serving as discovery tools to identify new candidate pathogenic mechanisms, as a means of testing hypotheses arising from human studies, and as pre-clinical models in which to assess potential therapeutic strategies (2). There are multiple different experimental perturbations known to trigger NAFLD in animal models, which have been used to generate all components of the NAFLD spectrum (3–10). Such perturbations include genetic manipulation (e.g., ob/ob mouse), pro-steatotic or pro-inflammatory diets [e.g., methionine–choline deficient (MCD) diet], and toxic insults (e.g., streptozotocin injection). Combinations of these may be used to reflect the multifactorial nature of the human disease (e.g., ob/ob mice fed a MCD diet); however, a detailed discussion of rodent diets is beyond the scope of this review.

While animal models do potentially give key insights into the disease process that cannot be obtained through any other means, it is necessary to temper appreciation of this utility with understanding of the limitations of each of the models used.

We, now, briefly review the current understanding of the genetic architecture of human NAFLD, and the major approaches adopted to modeling it in animals, while critically appraising the utility of these models.

## Overview of Non-Alcoholic Fatty Liver Disease Pathogenesis

Human NAFLD is defined by the presence of macrovesicular steatosis in more than 5% of hepatocytes in individuals with a history of less than 20 g/day ethanol intake (11). The differentiation of macrovesicular from microvesicular steatosis is a qualitative histological assessment, and the two may coexist in more advanced disease (12). NAFLD exists as a pathological spectrum ranging from non-alcoholic fatty liver (NAFL, also known as “simple” steatosis), which denotes hepatic steatosis in the absence of steatohepatitis, through to non-alcoholic steatohepatitis (NASH), in which there is histological evidence of inflammatory infiltrates, hepatocyte ballooning (featuring Mallory-Denk bodies), and commonly fibrosis of varying severity. Cirrhosis can develop at the most severe end of the spectrum, conferring increased risk of hepatocellular carcinoma (HCC), portal hypertension, and liver failure (13). Longitudinal studies have shown 10–20% of patients with each stage of disease to progress to the following stage in, while there is also an element of reversibility, particularly in NAFL and NASH (14, 15).

A “two-hit hypothesis” has been suggested to account for the pathogenesis of advanced NAFLD, whereby the first “hit” drives development of steatosis and the second triggers inflammation and its sequelae, critically including fibrosis (16).

**Figure 1:**
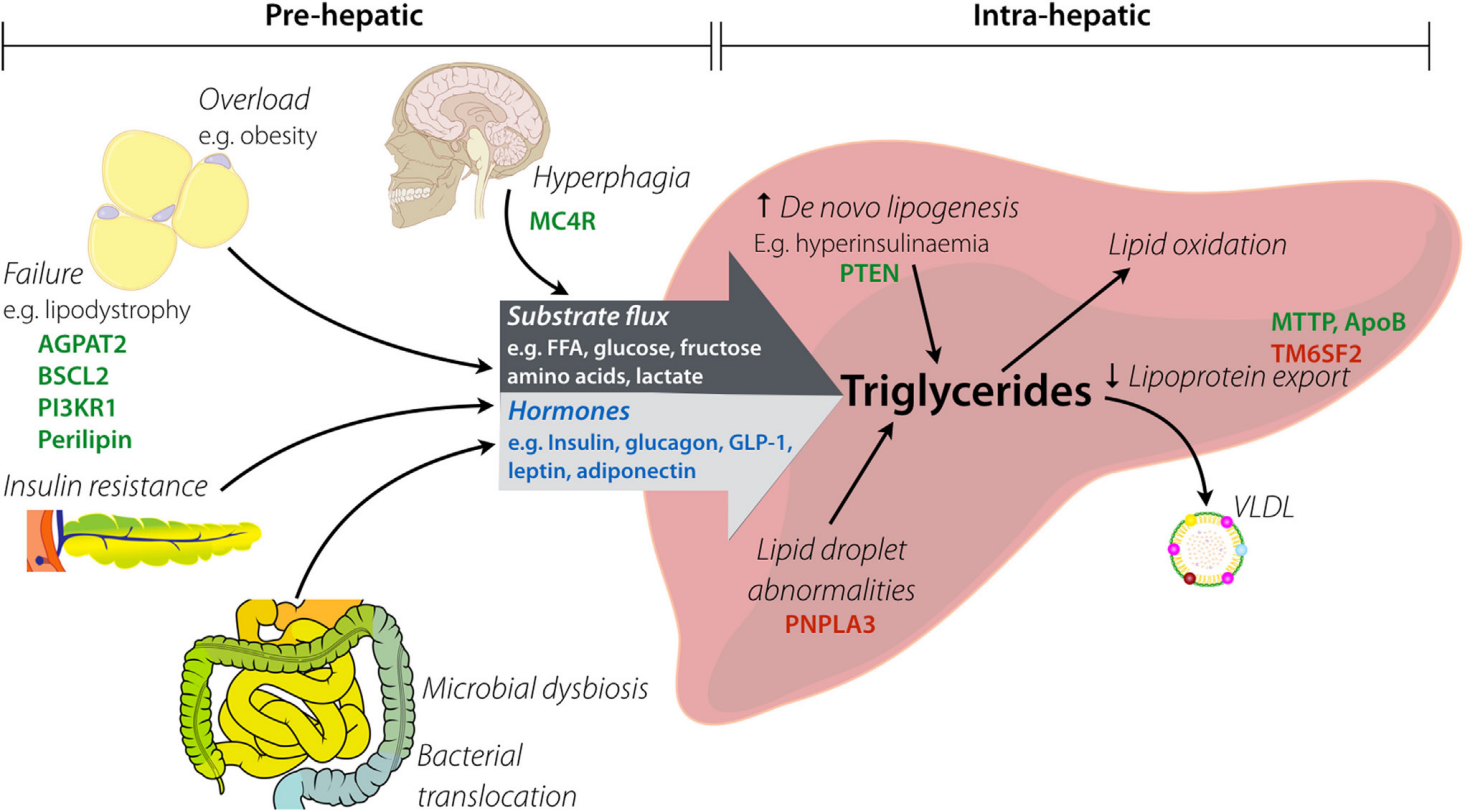
**Factors implicated in hepatic steatosis**. Pre-hepatic factors lead to increased substrate flux to the liver by both increased load (e.g., raised fatty acid delivery) and altered composition (e.g., increased fructose). Hyperphagia causes provision of excess macronutrients, which contributes to overload of white adipose tissue (*via* obesity). Lipodystrophy causes functional or anatomical failure of adipose, with the resulting spill over of substrates passing to the liver. Insulin resistance contributes to hormonal changes (e.g., raised insulin, low adiponectin) that alter intra-hepatic metabolism of lipids. Intestinal dysbiosis influences both substrate delivery to the liver and generation of gut-derived hormones (e.g., elevated GLP-1). Key: hormones are in blue, examples of genes involved in monogenic disorders are in green, and examples of genes with pro-steatotic common polymorphisms are in red.

In principle, hepatic steatosis may result from pre-hepatic derangements, intra-hepatic derangements, or both, as illustrated in simplified form in Figure 1. A comprehensive review of pathways leading to hepatic steatosis is beyond the scope of this article and is provided elsewhere (17–19). In brief, pre-hepatic pathogenic factors encompass increased substrate flux to the liver (e.g., non-esterified free fatty acids (NEFA), monosaccharides, or amino acids) and dysregulation of hormones that act directly on hepatocyte metabolism (e.g., insulin, glucagon and related peptides, and adipokines). Increased “preload” in the form of NEFA delivery generally results from failure of adipose tissue adequately to buffer positive energy balance. This may be a consequence of hyperphagic obesity, in which even normal adipose buffering capacity is overwhelmed, or lipodystrophy, in which adipose energy buffering capacity is pathologically constrained. In some situations, a mixture of these is at play, as in generalized lipodystrophy, where the harmful results of absent adipose tissue are potentiated by concomitant lack of leptin.

Many pre-hepatic hormonal factors also influence propensity to NAFLD by acting on adipocytes to modulate lipolysis and/or through direct actions on hepatocytes (e.g., insulin, glucagon, glucagon-like peptides). There has been a particular focus on the ability of high levels of insulin, secondary to peripheral insulin resistance, to drive hepatic *de novo* lipogenesis. Another emerging influence on liver metabolism is the gut microbiome, which may affect gut hormone release and also signal directly through flux of bacterial metabolites such as acetate (20, 21).

Hepatocyte-autonomous (intra-hepatic) defects may also lead to triglyceride accumulation. Such defects may broadly be classified into: those increasing *de novo* synthesis of triglyceride; those perturbing lipid droplet dynamics, triglyceride mobilization and lipoprotein assembly or secretion; and those impairing catabolism of fatty acids by beta-oxidation. Although reduced ability to catabolize fatty acids *via* beta-oxidation (e.g., due to Mendelian disorders in key catabolic enzymes, or mitochondrial dysfunction) does result in hepatic steatosis, however, this is usually microvesicular in appearance and has a distinct clinical profile that often includes hypoglycemia, liver failure, and encephalopathy (22). These disorders will, thus, not be discussed further here.

Development of NASH is multifactorial; a comprehensive review of the inflammatory and fibrotic sequelae of hepatic lipid accumulation can be found elsewhere (23–26). Key elements of pathogenesis include oxidative stress (from lipid peroxidation and mitochondrial dysfunction) and activation of pro-inflammatory pathways (e.g., NF-κB) in hepatocytes, but other cellular pathways, including the endoplasmic reticulum stress response, have also been implicated (27). Coactivation of Kupffer cells, sinusoidal endothelium, and hepatic stellate cells gives rise to cytokines that augment inflammation [e.g., tumor necrosis factor alpha (TNFα), interleukin-1/-6] and drive fibrosis [e.g., transforming growth factor beta (TGFβ)] (19, 28, 29). These processes are also exacerbated by pre-hepatic factors, such as adipose inflammation/lipotoxicity, gut bacterial translocation, and endogenous alcohol production.

## Human Genetics of NAFLD

In the vast majority of patients, NAFLD is a multifactorial condition rooted in obesity and insulin resistance, based on strong clinical association and natural history studies in humans. Pandemic, idiopathic NAFLD is often referred to as “primary” NAFLD (30). Genetics can play a role in each stage of the pathophysiology of NAFLD, as illustrated both by rare monogenic conditions that feature severe NAFLD, and by the association of much more frequent single nucleotide polymorphisms (SNPs) with “common” NAFLD (31–33). The proliferation of recent human genetic findings puts their detailed treatment beyond the scope of this discussion; however, we select a series of mechanistically informative sentinel examples to appraise against rodent models.

## “Pre-Hepatic” NAFLD

### Monogenic Hyperphagic Obesity

Flux of substrates, such as free fatty acids, amino acids, and lactate, provide the building blocks for hepatocyte triglyceride accumulation as well as the energy required for activation of anabolic pathways (see Figure 1). Excess flux can thus be a potent driver for NAFLD. Most attention has been paid to flux of free fatty acids, the product either of lipolysis of triglyceride in adipose tissue or lipolysis of triglyceride in triglyceride-rich lipoproteins such as chylomicron remnants. Key determinants of free fatty acid flux to the liver are thus the dietary intake of fat and the efficiency of fatty acid trapping and storage in adipose tissue. Correspondingly, in states of hyperphagia or adipose tissue insufficiency, free fatty acid flux may be pathologically increased. Of note, “adipose tissue insufficiency” may arise from overload of adipose tissue that is normal or increased in amount, or may arise from frank anatomical deficiency of adipose tissue, or lipodystrophy.

Several human examples of monogenic hyperphagia exist. The first to be discovered was congenital leptin deficiency (34), with loss-of-function mutations in the leptin receptor gene discovered later (35). Leptin is an adipose-derived peptide adipokine that acts on the hypothalamus to signal replete energy stores and suppress appetite (36). It is expressed and secreted by white adipose tissue (WAT) in direct proportion to WAT volume, and serum levels fall commensurately with depletion of adipose stores during starvation. Primary leptin deficiency produces severe, hyperphagic obesity from a young age; however, liver fat accumulation has only rarely been commented on in published studies. In one study, severe NAFLD was identified in an obese leptin-deficient child that resolved quickly on introduction of recombinant leptin therapy (37). The long-term hepatic outcome of untreated human leptin deficiency or leptin receptor mutation is not known; however, leptin receptor polymorphisms have been associated with NASH and insulin resistance in patients with NAFLD (38, 39).

Several other forms of human monogenic obesity are now known. In contrast to genetic leptin or leptin receptor loss-of-function, which are extremely rare, heterozygous mutations in the melanocortin-4-receptor (MC4R) are the most common monogenic cause of obesity, accounting for around 6% of severe, early-onset obesity (40). There are no published reports of the impact of heterozygous MC4R mutations on hepatic steatosis in monogenic obesity, while polymorphisms in MC4R have been associated with alanine aminotransferase and BMI (41), but not with hepatic fat content in population-wide studies (42). Several other genes have been implicated in human monogenic obesity; however, in aggregate, these affect only a very small number of patients, and there is little information on the liver phenotype. Moreover, some of the gene products involved, such as prohormone convertase 1 and pro-opiomelanocortin have pleiotropic roles, not just in appetite control but also in the action of key peripheral hormones (peptide and steroid hormones respectively) which have independent effects on liver fat accumulation, so shall not be considered here.

The sparse attention paid in published human reports to the natural history of NAFLD in human monogenic obesity is likely to be attributable to the rarity of the diseases, to the fact that most patients identified are children in whom clinically overt liver NAFLD has not yet had time to develop and to that fact that, in leptin deficiency, curative therapy with recombinant human leptin is the standard of care. This means that the long-term natural history of NAFLD in leptin deficiency will be extremely difficult to document in future, although determining the long-term liver outcome of MC4R loss-of-function should be both tractable and informative.

### Monogenic Lipodystrophy

In the face of severe hyperphagia, the ability even of “normal” adipose tissue to buffer chronic positive energy balance by trapping and sequestering lipid is finite and is eventually overwhelmed, leaving the liver exposed to excess free fatty acid flux. However, some rare humans have a congenital deficiency of adipose tissue, which we shall consider only in its most severe, generalized form. In this situation, there is effectively absolute adipose failure, and complete lack of energy buffering is compounded by secondary lack of leptin and consequent hyperphagia, as the hypothalamus erroneously interprets very low or absent leptin as an indication of starvation. Around 95% of cases in humans congenital generalized lipodystrophy (CGL) are accounted for by biallelic mutations in either AGPAT2, encoding an enzyme involved in triglyceride synthesis, or in BSCL2, encoding an endoplasmic reticulum protein involved in lipid droplet regulation and adipocyte differentiation (43, 44). A clinical hallmark of CGL is very severe NAFLD, with early development of inflammation, fibrosis, and HCC. Indeed, clinical experience and published case series suggest that complications of advanced liver disease are among the major sources of mortality in CGL (45, 46). Observations in CGL and other acquired and genetic lipodystrophies establish unequivocally that primary disorders of adipose tissue are sufficient to cause the full spectrum of NAFLD in humans (47). This does not prove that adipose dysfunction is the primary mechanism at play in pandemic NAFLD; however, current population-wide data do not rule this out.

### Endocrine Drivers

Many hormones exert a major influence on lipid accumulation within hepatocytes, although in each case this is only possible in the context of adequate energy charge and substrate flux to the liver, so these cannot easily be teased apart. Hormones of proven importance in altering liver lipid accumulation include insulin, glucagon, and the incretin gut peptides (e.g., GLP-1). In humans, insulin has attracted particular attention as several lines of evidence suggest that insulin, acting through the insulin receptor, stimulates *de novo* lipogenesis sufficiently to make a major contribution to liver fat accumulation in NAFLD (48–50). Some negative evidence in support of this model comes from the observation that humans with loss-of-function mutations in the insulin receptor itself, although showing very severe insulin resistance, are protected from dyslipidemia and liver fat accumulation (51). Preliminary evidence suggests that this also holds for patients with lipodystrophy and loss-of-function mutations in *PIK3R1*, a component of phosphatidylinositol-3-kinase involved in insulin signaling (52).

## Hepatocyte-Autonomous NAFLD

### Lipid Trafficking Abnormalities

Some genetic abnormalities can influence development of steatosis by hepatocyte-autonomous mechanisms. One of these mechanisms is impaired assembly and export of lipoproteins from hepatocytes, leading to intracellular accumulation of triglycerides. The best examples of this are heterozygous truncating mutations in APOB [encoding apolipoprotein B (ApoB)], causing hypobetalipoproteinaemia and biallelic mutations in MTTP (encoding microsomal triglyceride transfer protein), which cause abetalipoproteinaemia (53). These two conditions have a similar phenotype including NASH, low serum triglycerides, low LDL cholesterol, and neuropathy. Patients often develop progressive NASH with fibrosis even in the absence of diabetes, obesity, or other oxidative stresses (53, 54).

Further evidence in support of altered lipoprotein assembly and secretion as a significant player in NAFLD comes from GWAS of the commoner form of the disease. These have implicated a SNP in TM6SF2 (encoding transmembrane 6 superfamily member 2), which is needed for secretion of very-low density lipoproteins (VLDL), in NAFLD. The risk allele is found in around 1% patients with NAFLD (55) and correlates positively with serum aminotransferase levels (33), hepatic steatosis (31), NASH activity, and fibrosis (but not HCC) (56). Importantly, patients with this SNP have lower circulating LDL cholesterol, lower triglycerides, and reduced incidence of atherosclerotic disease, providing an example of dissociation of NAFLD from dyslipidemia and cardiovascular disease (57). Data relating to the association with insulin resistance and type 2 diabetes are currently inconclusive (58).

Population-wide human genetics has also implicated fundamental abnormalities in lipid droplets in NAFLD: for example, the p.Ile148Met SNP in PNPLA3, whose product is patatin-like phospholipase domain-containing 3, is associated with all stages of NAFLD, from simple steatosis, NASH, fibrosis, and development of HCC (59, 60). PNPLA3 (also known as adiponutrin) is a membrane-bound enzyme expressed at the surface of lipid droplets and on the smooth endoplasmic reticulum (61) and plays a role in lipid droplet dynamics. There has been conflicting evidence on whether this polymorphism is associated with insulin resistance, however.

### Inflammation/Fibrosis

Inflammation and fibrosis are the hallmarks of complicated NAFLD, and a genetic basis for the susceptibility to these complications is often mooted as the “second hit” needed to drive progression from simple steatosis to inflammatory and fibrotic sequelae. Unsurprisingly, no single gene examples of a primary inflammatory or fibrotic disorders leading to end stage NAFLD have been reported (62, 63); however, the GWAS approach has identified common genetic variants associated with progression of fibrosis (e.g., near the gene for platelet-derived growth factor alpha) or with the development of lobular inflammation (e.g., near genes encoding interleukin-6, or collagen type XIII alpha 1).

## Rodent Genetic Models of NAFLD

The ideal “one stop” animal model of NAFLD would recapitulate the full progression from simple steatosis through NASH to fibrosis, cirrhosis, and HCC. Ideally, the disease process would progress rapidly (in contrast to the human condition) to maximize experimental tractability of the model. However, a valid alternative would be to have a series of different models for different stages of the disease sequence, which may be primarily metabolic, inflammatory, or fibrotic. It has been suggested that the primary goal of rodent models should be to mimic the pathology of NAFLD, including the underlying histopathology (3). On this narrow basis, it has been argued that rodents do not accurately model human NAFLD because of differences in ballooning degeneration and distribution of inflammatory infiltrate in NASH (4).

Many animal models have been described in which different degrees of NAFLD are seen, and we shall consider selected models only as examples, classifying them into two groups according to the broad pathogenic mechanism at play: (1) models of “extrahepatic” NAFLD (Table 1) and (2) models of “intrahepatic” NAFLD (Table 2).

**Table 1:** **Examples of murine genetic models relevant to “pre-hepatic” NAFLD**.

Model	Obesity	Insulin resistance	Hyper-lipidemia	Liver steatosis	NASH	Fibrosis	HCC
**Hyperphagic models**							
*Ob/ob* (64)	Y	Y	Y	Y	Y	Y (mild)	?
*Db/db* (64)	Y	Y	Y	Y	Y	Y (mild)	?
Mc4r^−/−^ on HFD (65)	Y	Y	Y	Y	Y	Y	Y
**Lipodystrophic models**							
Agpat2^−/−^ (66)	N	Y	N	Y	?	?	?
Bscl2^−/−^	N	Y	N	Y	?	?	?
A-ZIP/F-1 (67)	N	Y	Y	Y	?	?	?
Adipose-specific *Insr* knockout (68)	N	Y	Y	Y	Y	Y	Y
**Liver insulin action**							
Liver-specific *Pten* knockout (69)	N	N	N	Y	Y (mild)	Y	Y

*Features are based on *ad libitum* standard chow diet, unless otherwise stated. *ob/ob* is a leptin-deficient mouse; *db/db* is a leptin-resistant mouse; MC4R^−/−^ mouse is a mouse with hypothalamic hyperphagia; Agpat2^−/−^ and Bscl2^−/−^ are lipodystrophic mice with functional and anatomical failure of white adipose; A-ZIP/F-1 is a lipodystrophic mouse due to specific failure of white adipose differentiation; adipose-specific insulin-receptor (Insr) knockout causes lipodystrophy; and liver-specific phosphatase and tensin homolog (PTEN) knockout disrupts hepatocyte insulin signaling*.

*HCC, hepatocellular carcinoma; HFD, high-fat diet; NASH, non-alcoholic steatohepatitis; Y, yes; N, no; ?, insufficient data*.

**Table 2:** **Examples of murine genetic models relevant to “intrahepatic” NAFLD**.

Model	Obesity	Insulin resistance	Hyper-lipidemia	Liver steatosis	NASH	Fibrosis	HCC
**Impaired lipoprotein synthesis/secretion**							
*Apob*^−/−^ (70)	N	N	Y	Y	Y	Y	N
Fatty liver Shionogi (FLS) (71)	N	Y	N	Y	Y	Y	Y
FLS-*ob/ob* mouse (72)	Y	Y	Y	Y	Y	Y	Y
*Tm6sf2* knockdown (32)	N	Y	Y	Y	N	N	N
**Abnormal lipid droplet dynamics**							
Hepatic-specific *PNPLA3* I148M expression (73)	N	Y	N	Y	N	N	N

*Features are based on *ad libitum* standard chow diet. *Apob*^−/−^ mouse is unable to secrete VLDL from hepatocytes; fatter liver Shionogi mice are thought to also have impaired VLDL secretion; FLS-*ob/ob* are FLS mice crossed with leptin-deficient mice; Transmembrane 6 superfamily 2 (Tm6sf2) knockdown mice have reduced triglyceride secretion; hepatic-specific expression of the human I148M allele of patatin-like phospholipase domain-containing 3 (PNPLA3) causes altered lipid droplet composition and storage*.

*NASH, non-alcoholic steatohepatitis; HCC, hepatocellular carcinoma; Y, yes; N, no*.

## Murine “Pre-Hepatic” NAFLD

### Hyperphagic Models

The severely obese *ob/ob* mouse strain arose spontaneously in 1949, and was eventually discovered in 1994 to harbor a loss-of-function mutation in the gene encoding leptin, thereby ushering in the modern era of investigation of the neuroscience of appetite control. Given an *ad libitum* diet, *ob/ob* mice develop obesity, insulin resistance, hyperglycemia, and hepatic steatosis (74), and they have been become a highly popular model for many aspects of obesity and related disorders, either being studied in isolation, or crossed with other strains of interest in order to determine the effect of severe hyperphagia on mice with different genetic perturbations. The severe obesity of *ob/ob* mice is attributable to hypothalamic hyperphagia; however, several studies have provided evidence for additional peripheral effects of leptin to modulate insulin sensitivity and metabolism directly in tissues, such as muscle and liver. Whether such peripheral effects are also seen in humans remains to be established beyond doubt (75–77).

The *ob/ob* mouse rapidly develops NAFL and at 20 weeks old early features of NASH are present (77, 78). NASH may been accelerated by a second “hit” of inflammatory/oxidative stress, using, for example, intraperitoneal injection of lipopolysaccharide (LPS) (79), dietary stressors such as a MCD diet (80), or crossing with another NASH-prone strain, such as the fatty liver Shionogi (FLS) mice, which are discussed later (81). Notably, however, leptin is required for normal immune function, and its deficiency dampens down both innate and acquired immune responses in humans (82, 83), though it seems to play a pro-inflammatory and pro-fibrogenic role in mice (78). This may explain why the inflammatory components of NAFLD appear relatively indolent in *ob/ob* mice than in more common NAFLD. On a related note, it has also be shown that the *ob/ob* mouse is relatively resistant to fibrosis (78, 80), partly due to reduced release of TNFα, which is necessary for activation of TGFβ, a key pro-fibrogenic molecule (84–86).

Collectively, these findings caution that, while the *ob/ob* mouse is a valuable model of primary hyperphagia-driven NAFLD, loss of specific actions of leptin on peripheral metabolism, coupled to some degree of immunosuppression, means that it is likely to deviate from pandemic NAFLD in key respects, especially related to the inflammatory and fibrotic end of the disease spectrum. Similar arguments may apply to leptin-resistant *db/db* mice, which harbor a splice site mutation abolishing expression of the long form of the leptin receptor; however, *db/db* mice are reported to exhibit more severe NAFLD than *ob/ob* mice (64, 80). In practice, a secondary stressor is also usually applied when using *db/db* mice to study NAFLD, with the MCD diet being widely used (87).

Mice in which *Mc4r* is genetically ablated have also been widely studied. Their primary defect lies within hypothalamic appetite control pathways, as the melanocortin 4 receptor responds to the neuropeptide α-MSH, which is generated in response to leptin action. *Mc4r*-null mice feature hyperphagic obesity without pathologically suppressed leptin levels, suggesting that they have the potential to model the extended spectrum of NAFLD more faithfully than *ob/ob* mice. In keeping with this, it has been reported that *Mc4r*-knockout mice, when exposed to a high-fat diet, develop not only steatosis but also exuberant NASH with established fibrosis after 20 weeks, progressing to HCC in all mice studied by 1 year (65).

### Lipodystrophic Models

Many different murine genetic models of lipodystrophy have been described. Where direct comparison is possible between mice and humans, it has been found that the common forms of CGL are well modeled in mice, while the situation for human genetic forms of partial lipodystrophy is more complex, and will not be discussed further here (45).

Both *Agpat2*- and *Bscl2*-knockout mice have been described (88, 89). *Agpat2* encodes 1-acylglycerol-3-phosphate O-acyltransferase 2 and is needed for synthesis of triacylglycerols and glycerolphospholipids in white adipose. Many Agpat2^−/−^ mice die in the first few weeks of life; however, the survivors accurately recapitulate the human condition of lipodystrophy with severe insulin resistance (66). Hepatic steatosis has also been reported to be a major feature of the mice before 16 weeks old, with liver weights twice normal, and modest inflammatory changes seen in addition to pronounced steatosis. More detailed time course studies of the liver disease in this model have not been reported, however.

Bscl2 encodes seipin, which is needed for both differentiation of white adipocytes and normal lipid droplet regulation, and deficiency has been suggested to shift the balance toward release of free fatty acids from adipocytes (89). Bscl2^−/−^ mice display a lipodystrophic phenotype with near complete absence of WAT. Massive hepatic steatosis is a feature of all three Bscl2^−/−^ models described. As in Agpat2 null mice, however, detailed natural history studies of liver pathology have not been reported: at 12 weeks, there is no evidence of NASH but there are no reported data beyond this age (90).

Although not directly modeling human disease, some other murine models have provided powerful evidence for the importance of adipose tissue in protection from NAFLD. One impactful model is the A-ZIP/F-1 (or “AZIP”) mouse, which is an adipose-deficient mouse generated by transgenic overexpression of an artificially engineered dominant negative protein that interferes with critical adipogenic transcription factors. This causes deficiency of white adipose, severe insulin resistance, and hepatic steatosis, although it was said not to feature inflammation at the relatively young age studied (67). It is not known whether these mice develop NAFLD-related fibrosis, in part because of the reduced survival of the mice, which are severely diabetic. A much more recent model featured knockout of the insulin receptor selectively in adipose tissue, knockout being mediated by *cre* recombinase driven by the adipose-specific adiponectin promoter (68). These mice developed severe lipodystrophy and fatty liver disease very early, and by 12 weeks old, livers demonstrated not only steatosis but also increased ROS, lipid peroxidation, ballooning degeneration of hepatocytes, and elevated serum transaminase levels. By 1 year old the liver accounted for 25% of body weight and showed highly dysplastic liver nodules in addition to worsened inflammation and fibrosis. High fat feeding worsened the liver injury.

### Liver Insulin Action

Although it is impossible to mimic a primary increase in insulin without inducing complex and potentially confounding changes to systemic metabolism, liver-specific knockout of the insulin receptor does afford the opportunity to test the proposition that liver insulin action is necessary for the development of hepatic steatosis. Indeed, in 2008, it was reported that ablation of the insulin receptor in mouse liver, although inducing severe systemic insulin resistance, did not increase liver triglyceride or liver weight (91).

Conversely, liver-specific knockout of the phosphatase and tensin homolog (PTEN), which is a phosphatidylinositol-3,4,5-triphosphate (PIP_3_) phosphatase that serves to antagonize insulin’s metabolic actions, produces macrovesicular steatosis, NASH (including Mallory-Denk bodies), fibrosis, and HCC (69). Similarly, other modes of genetic activation of phosphatidylinositol-3-kinase pathway signaling also produce steatosis that progresses to HCC, but without such marked steatohepatitis (92, 93).

## Hepatocyte-Autonomous Murine NAFLD

### Impaired Lipoprotein Synthesis/Secretion

Assembly and secretion of VLDL represents a major route for the final disposition of intrahepatocyte triglyceride. Apolipoprotein B (ApoB) is a core component of VLDL particles, and genetic partial deficiency in humans causes familial hypobetalipoproteinaemia. Homozygous loss-of-function mutations of ApoB in mice cause embryonic lethality due to exencephalus. However, several different lines of mice heterozygous for mutated ApoB have been described, and these accurately recapitulate the hepatic steatosis, low serum triglycerides, and low HDL cholesterol of the human condition, although little fibrosis has been reported in the absence of additional pro-inflammatory stimuli (70, 94). Nevertheless, an important caveat is that natural history of liver inflammation and fibrosis has not been reported beyond 12 weeks of age. Similar to humans with hypobetalipoproteinaemia, mice with reduced ApoB function do not show marked insulin resistance, which differentiates them from most patients with NAFLD.

The FLS mouse is a further NAFLD model, which arose spontaneously as a result of inbreeding. FLS mice are non-obese and only mildly insulin resistant, but show marked accumulation of macrovesicular triglyceride with mononuclear inflammatory infiltrate and fibrosis, which eventually results in development of HCC by 13–16 months even without additional carcinogenic stimuli (95, 96). The precise underlying genetic defect is not yet known; however, a defect in microsomal triglyceride transfer protein (MTTP) (97) has been suggested. Precisely, why this model is so susceptible to HCC is not known. Crossing of FLS mice with *ob/ob* mice results in a model with the full metabolic syndrome and progressive fibrosis (81, 98); this model most closely recapitulates the whole human spectrum of NAFLD in a practical, experimental timeframe.

As discussed, human GWAS have shown a SNP in TM6SF2 to be associated with NAFLD (99). Reminiscent of hypobetalipoproteinaemia, however, carriers of the risk-conferring T-allele seem relatively protected from atherosclerotic disease despite increased lipid fat content. TM6SF2 is a membrane-bound protein located on the endoplasmic reticulum involved in the secretion of VLDL from hepatocytes (100). Germline knockout of *Tm6sf*^−^ has not yet been described in mice; however, adeno-associated virus-mediated knockdown selectively in mouse liver resulted in increased hepatic triglyceride content and reduced VLDL secretion (32). Moreover transgenic hepatocyte-specific expression of human wild-type TM6SF2 in mice caused an increase in serum LDL cholesterol and lower HDL cholesterol (101); however, the full spectrum of NAFLD has not been demonstrated in this model.

## Abnormal Lipid Droplet Dynamics

The p.Ile148Met variant in human PNPLA3, found in around 20% of the population, is associated with NAFLD progression (55). Therefore, efforts have been made to generate a rodent model that recapitulates this genetic predisposing factor and to elucidate the role of PNPLA3 in NAFLD. PNPLA3 is expressed in many tissues in mice, including WAT and liver (102). Expression is suppressed upon fasting (103) and upregulated following a carbohydrate load (104), which is believed to be mediated by steroid regulatory element-binding protein-1c (SREBP-1c) and carbohydrate-response element-binding protein (ChREBP). PNPLA3 was initially thought to function as a lipase and be involved in the release of triglycerides from intracellular lipid droplets (105–107), so it was hypothesized that reduced activity would increase hepatocellular triglyceride content (108).

If reduced PNPLA3 activity was to accelerate NAFLD, *Pnpla3* knockout mice would be expected to have severely fatty livers; however, in fact, they have no evidence of NAFLD (109). Indeed, there is no discernible difference between *Pnpla3*^−/−^ and wild-type mice even when fed high-fat diet or crossed onto the *Lep^ob/ob^* background. It should be noted, however, that mice show the highest Pnpla3 expression in WAT, unlike humans, in whom expression is highest in the liver.

In contrast to the knockout mice, mice with hepatic overexpression of human PNPLA3 p.Ile148Met show increased hepatic triglyceride content and fatty acid synthesis (73, 110). They develop steatosis only on a normal chow or sucrose diet, but not on a high-fat diet, which suggests that the mutant PNPLA3 acts upon *de novo* fatty acids, rather than re-absorbed circulating non-esterified fatty acids (111). This is consistent with epidemiological data that suggests fructose-rich diets are more harmful than high-fat diets (112).

The cumulative data from these mouse models suggest that PNPLA3 functions as a lysophosphatidic acid acyltransferase, and that the p.Ile148Met polymorphism increases this activity, stimulating development of hepatic steatosis (113, 114). This would have not been identified without the initially surprising finding in the knockout model. Although mice with hepatic expression of human PNPLA3 p.Ile148Met develop NAFLD, it is not known whether these mice develop HCC with age as reported studies extend to only 12 weeks, however.

## Conclusion

The spectrum of human NAFLD disease arises from complex environmental influences allied to genetic predisposition. Human monogenic diseases demonstrate unequivocally that adipose tissue failure and primary genetic defects in intrahepatocyte lipid handling can give rise to NAFLD and its sequelae. Genetic perturbation in rodents may be combined with a pro-inflammatory or pro-steatotic diet to accelerate liver injury and mimic the multifactorial nature of human NAFLD. In general, murine genetic models closely mimic monogenic forms of NAFLD, where mouse and human liver phenotypes have been described in sufficient detail to draw conclusions; however, many gaps exist in descriptions of the natural history of NAFLD associated with several of the monogenic diseases in mice or humans. Findings in both species indicate that there is more than one possible pathogenic route to NAFLD, meaning that study of several different human conditions and their models will be important to tease out common mechanisms of liver damage. A critical advantage of mouse models over humans is that the tremendously powerful technologies available to perturb genes conditionally or in an organ-specific way permits isolation of only some parts of a highly interconnected system. So, while alignment of humans and their murine models could be further refined, murine models are a highly valuable tool in the study of NAFLD.

## Author Contributions

JM, RS, and MA were all involved in conceptualizing and writing the manuscript, in its critical review, and in approval of the final version.

## Conflict of Interest Statement

The authors declare that the research was conducted in the absence of any commercial or financial relationships that could be construed as a potential conflict of interest.
